# Prevalence of obesity and its associated factors among healthcare workers in Malaysia: a scoping review

**DOI:** 10.1186/s12889-026-27143-4

**Published:** 2026-04-14

**Authors:** Syakirah Roslan, Rozita Hod, Nora Saliza Md Salim, Azimatun Noor Aizuddin, Rosnawati Mohamad Robat, Mohd Fadhli Mohd Fauzi, Mohd Shahrol Abd Wahil

**Affiliations:** 1 Department of Public Health Medicine, Faculty of Medicine, Universiti Kebangsaan Malaysia, Jalan Yaacob Latiff, Bandar Tun Razak, Cheras, Kuala Lumpur, 56000 Malaysia; 2 Ministry of Health Malaysia, Block E1, E3, E6, E7 & E10, Complex E, Federal Government Administrative Centre, Putrajaya, 62590 Malaysia; 3 Selangor State Health Department, No. 1, Wisma Sunway, Jalan Tengku Ampuan Zabedah C 9/C, Section 9, Shah Alam, Selangor 40100 Malaysia

**Keywords:** Obesity, Overweight, Healthcare workers (HCWs), Blood pressure, Anxiety, Non-communicable disease, Prevalence, Malaysia

## Abstract

**Objectives:**

This scoping review sought to consolidate Malaysian research on excess body weight among healthcare workers by (i) mapping published prevalence estimates of overweight and obesity, and (ii) describing reported associated risk factors within four domains: sociodemographic and occupational, behavioural, psychosocial, and anthropometric.

**Methods:**

PubMed, Scopus, and Web of Science (WoS) were searched for records published between January 2000 and July 2025. Empirical reports and completed systematic reviews that presented prevalence estimates or determinants of overweight or obesity among Malaysian healthcare workers were eligible. Eight studies met these criteria. Data on study setting, sample size, diagnostic thresholds for body mass index (BMI), prevalence, and effect sizes for risk factors were extracted and organised thematically. No meta-analysis was attempted because of methodological heterogeneity.

**Results:**

Across eight studies, combined overweight plus obesity prevalence ranged from 21.0% to 55.9%, consistently exceeding the national adult averages. Nursing, support, and administrative staff recorded the highest levels; one screening study showed that 63.0% to 100.0% of these staff categories were above the healthy weight range. Individual aged 40 years and older, employment duration of at least five years, and nurse job title each increased the likelihood of obesity by roughly twofold. Low physical activity, meal skipping, sugared-drink intake and restrained eating style was reported in three studies, while depressive symptoms and short sleep was linked to higher weight. Two reports documented clustering of central adiposity with elevated blood pressure in younger nurses.

**Conclusions:**

Malaysian healthcare facilities were found to be as obesogenic workplaces, particularly for long-serving nursing, support, and administrative staff. Interventions should combine specific schedule reforms, healthy food access, movement-promoting design and integrated mental health support. These programmes should be evaluated using longitudinal metrics of body mass and cardiometabolic health.

## Key points

a) Overweight and obesity are recognised as a growing problem among healthcare workers worldwide, but Malaysian evidence is scattered across small, single-centre surveys that report widely differing prevalence figures and examine different risk factors.

b) By collating eight Malaysian studies, this scoping review shows that 21.0% to 55.9% of healthcare workers are above a healthy weight, with risk almost twice as high in nurses, long-serving staff, and those reporting low physical activity, meal skipping or depressive symptoms.

c) This synthesis identifies clear intervention targets, protected meal breaks, movement-promoting workspace design and integrated mental health support, and highlights the need for longitudinal surveillance and tailored staff wellness programmes to curb obesity within Malaysia’s healthcare system.

## Introduction

Obesity is a complex chronic disease that affects all stages of life [1] and can have serious consequences on health [2], quality of life and economic outcomes. It is now the leading contributor to non-communicable diseases in adults globally [3]. Obesity is defined as having body mass index (BMI) exceeding 25.0 kg/m^2^ while overweight is defined as a BMI ≥ 23.0 kg/m² [4]. BMI is useful as a screening tool at the individual level and for estimating population-level obesity [5].

Recent study shows that more than one billion people worldwide are now living with overweight or obesity [6]. Obesity among adults and working-age population has doubled since 1990 and continues to rise, reaching 43.0% in 2022 [6]. Healthcare workers (HCWs) are no exception to this growing burden [7]. Despite having to be role models to increase community awareness related to disease prevention and advocacy, several studies have shown upward trend among HCWs towards obesity and overweight.

In Malaysia, the Ministry of Health introduced the KOSPEN WOW programme [8] to address non-communicable diseases, including obesity, among government HCWs and selected or volunteer agencies. KOSPEN WOW is an acronym for *Komuniti Sihat Pembina Negara* (KOSPEN) Wellness of Workers (WOW). The main objective of the KOSPEN WOW programme is to cultivate healthy and productive employees by fostering a conducive and supportive work environment that promotes a healthy lifestyle [9].

Various studies across the world have reported a high prevalence of obesity [10]. In Malaysia, several studies have examined the prevalence of obesity among HCWs [11, 12]. A recent study conducted in year 2021 in the east coast region of Peninsular Malaysia involving government HCWs showed that 21.1% of them were obese. Many of these studies focus on HCWs in district health office due to their accessibility. This setting is logistically simpler and more economical compared to hospitals. Moreover, district health office populations are often more manageable for initial studies, making data interpretation more feasible.

Obesity among HCWs has become a recognised occupational and public health concern in Malaysia [12], yet the evidence base remains scattered across isolated hospital surveys, clinic-based audits, and screening reports. Collectively, these studies suggest that excess body weight is common in the health sector and that its drivers extend beyond individual lifestyle choices [13] to include work schedules, job roles, psychological strain and emerging cardiometabolic risks. Because the findings are dispersed and their methodological approaches differ, a clear, consolidated picture has not been available to policymakers or hospital administrators seeking to design targeted wellness initiatives [14].

Obesity among Malaysian HCWs has been investigated in many individual studies, yet the findings remain scattered and sometimes contradictory. To understand the breadth of evidence and identify where new data or interventions are most needed, we undertook a scoping review with two specific aims: (i) to collate and map all published estimates of overweight and obesity prevalence among Malaysian HCWs, and (ii) to critically examine the reported determinants—sociodemographic, occupational, behavioural, psychosocial, and biomedical—that have been linked to excess weight in this workforce. By synthesizing these disparate findings within a unified framework, the review provides a consolidated picture of the national burden of HCW overweight and obesity, highlights where knowledge gaps persist, and identifies which risk domains appear most amenable to targeted action.

## Methods

### Study design

This article was conceived as a scoping overview, following the integrative guidance of the PRISMA-ScR (Preferred Reporting Items for Systematic Reviews and Meta-Analyses extension for Scoping Reviews, 2018) checklist.

### Data sources and search strategy

To assemble the evidence base, the PubMed, Scopus, and Web of Science databases were searched for material published between 1 January 2000 and 26 July 2025. An assisted search strategy combined free text and controlled vocabulary across three concept blocks: (overweight OR obesity), (healthcare worker OR nurse OR doctor OR allied health) and (Malaysia OR Malaysian). The complete search string is provided in Table 1. Reference lists of all eligible articles and the National Health and Morbidity Survey (NHMS) 2023 report [15] were hand-searched, while forward citation tracking in Scopus and grey literature scans of OpenGrey and WHO IRIS captured additional sources.

**Table 1:** Complete search strategy for pubmed, scopus, and web of science

Database	Search string
PubMED	(“obesity“[MeSH Terms] OR “overweight*“[All Fields] OR “BMI“[All Fields]) AND (“health personnel“[MeSH Terms] OR ((“delivery of health care“[MeSH Terms] OR (“delivery“[All Fields] AND “health“[All Fields] AND “care“[All Fields]) OR “delivery of health care“[All Fields] OR “healthcare“[All Fields] OR “healthcare s“[All Fields] OR “healthcares“[All Fields]) AND “worker*“[All Fields]) OR “nurse*“[All Fields] OR “doctor*“[All Fields] OR (“personnel, hospital“[MeSH Terms] OR (“personnel“[All Fields] AND “hospital“[All Fields]) OR “hospital personnel“[All Fields] OR (“hospital“[All Fields] AND “staff“[All Fields]) OR “hospital staff“[All Fields])) AND (“malaysia“[MeSH Terms] OR “malaysia“[All Fields] OR “malaysia s“[All Fields] OR “malaysian*“[All Fields])
Scopus	TITLE-ABS-KEY ((“obesity" OR "overweight" OR "body mass index" OR "BMI”) AND (“healthcare workers" OR "healthcare professionals" OR "nurses" OR "doctors" OR "medical staff”) AND (“prevalence" OR "risk factors" OR "determinants" OR "associated factors”) AND (“Malaysia”))
Web of Science	(“obesity” [MeSH] OR overweight* OR BMI) AND (“health personnel” [MeSH] OR healthcare worker* OR nurse* OR doctor* OR hospital staff) AND (Malaysia OR Malaysian*)

The asterisk (*) is awildcard character used in search queries and databases to represent zero or more characters; for example, ‘worker’ may retrieve ‘worker’,‘workers’, or ‘workforce’

### Eligibility criteria and study selection

We included empirical studies (cross-sectional, cohort, mixed-methods) and completed systematic reviews that reported prevalence and/or determinants or overweight or obesity among practising Malaysian HCWs aged ≥ 18 years. Case reports, paediatric trainee studies, and papers focused solely on surgical or pharmacological treatments were excluded. Two reviewers independently screened titles, abstracts, and full texts, and disagreements were resolved by discussion.

### Data extraction and synthesis

For each eligible study, we recorded the author, year, setting, design, sample size, BMI cut-off, prevalence, risk factors examined, and effect sizes (adjusted odds ratios with 95% CI). We did not conduct a meta-analysis because the study designs and outcome metrics were heterogeneous. Instead, findings were collated into an evidence matrix and subjected to inductive thematic synthesis, organised into four domains: sociodemographic, occupational, behavioural, and psychological/organisational factors. These results are presented narratively in the text and summarised in tables and figures.

### Ethical approval and consent

Approval from a research ethics committee was not sought for this study because it is a synthesis of publicly available literature and contains no primary or individual-level data.

## Results

**Fig. 1:**
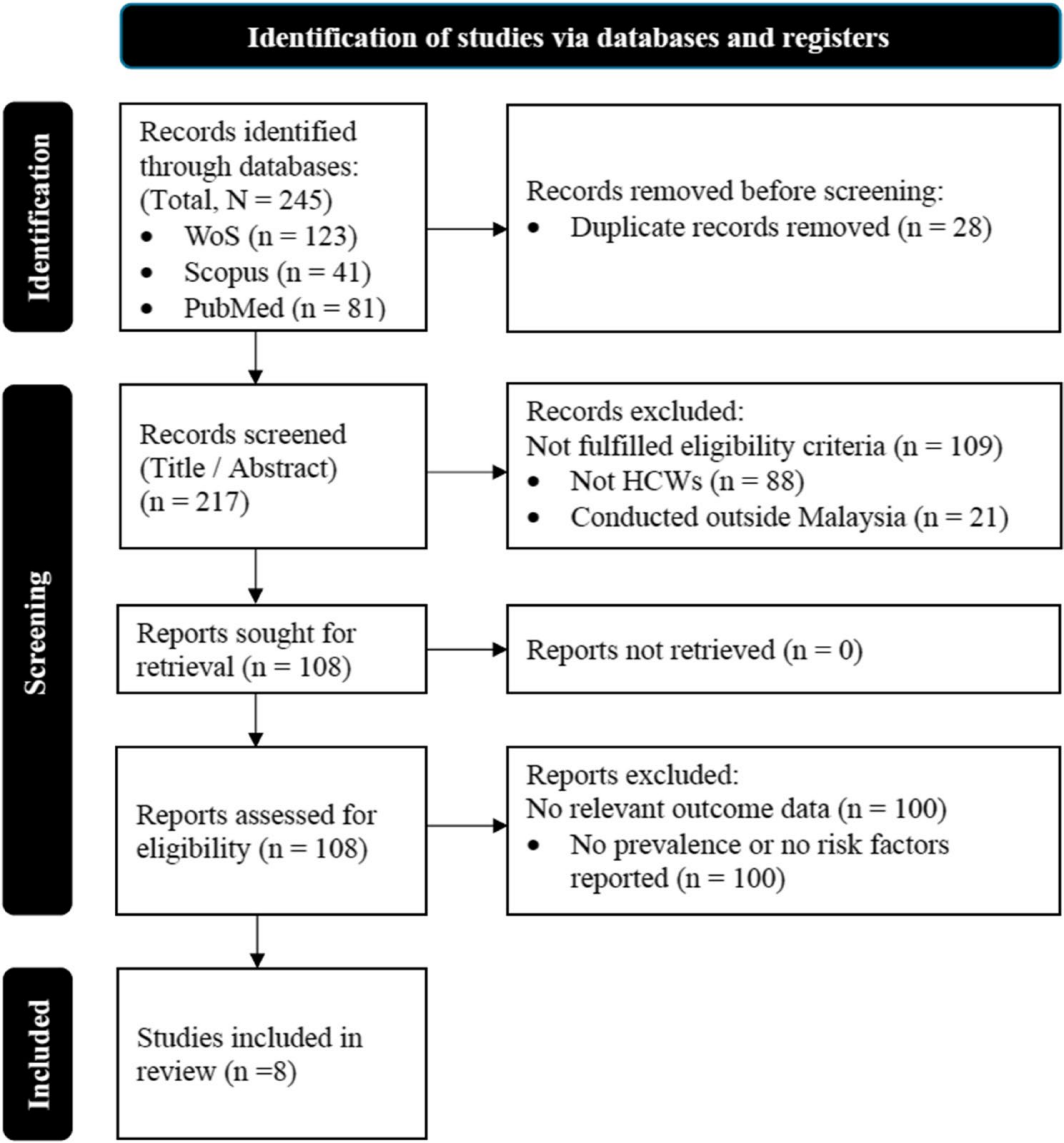
PRISMA 2020 flow diagram

The initial literature search identified 245 articles; after removing 28 duplicates, 217 articles remained for screening. Screening of titles and abstracts using the defined criteria led to the removal of 109 articles for the following reasons: not HCWs (*n* = 88) and studies conducted outside Malaysia (*n* = 21). Assessment for eligibility resulted in the removal of 100 studies due to the absence of relevant outcome data (*n* = 100). This left a final selection of eight articles for analysis in this scoping review. The study selection process is shown in Fig. 1.

### Characteristics of the included studies

The eight eligible studies were published between 2019 and 2023, with a noticeable rise after 2021; Six out of eight article were cross-sectional studies and approximately two-thirds sampled nurses. The median sample size was 280 participants (minimum–maximum = 80 and 4241).

### Characteristics of obesity/ overweight literature in Malaysia

The eight eligible studies yielded prevalence data from six of Malaysia’s thirteen states: Perak, Sabah, Kelantan, Terengganu and Pahang. However, none originated from the three federal territories (Kuala Lumpur, Putrajaya or Labuan). Perak contributed the highest number of single-site investigations, while Melaka, Sabah, and each of the east coast states were represented by one study each. No study provided state-specific obesity figures for Kuala Lumpur or the other federal territories, highlighting a persistent gap in the literature. The characteristics of the eligible studies in this review are presented in Table 2.

**Table 2:** Characteristics of the prevalence and risk factors of overweight and obesity (*N* = 8)

Author, Year	Study design	Study setting	Study population	Sample size	Diagnostic methods	Prevalence	Risk factors
Leow et al., 2022 [16]	Pre-post intervention	Simpang Health Clinic workforce (all staff)	HCWs in clinic	80	BMI ≥ 25 kg/m^2^, height / weight measured by trained personnel with calibrated stadiometers and scales	Combined 56.3% overweight / obese	Male; age 45–56 years old; job categories; drivers, admin clerks, health attendants, medical assistants
Kit et al., 2020 [17]	Cross sectional	12 primary-care clinics, Perak (mixed HCWs)	Mixed HCWs	261	BMI ≥ 25 kg/m^2^, height / weight measured by trained personnel	Combined 49.9% overweight / obese	Older age; professional/managerial grade; longer tenure associated with higher obesity indices; Poorer self-rated health linked to higer obesity indices
Kunyahamu et al., 2021 [11]	Cross sectional	Government HCWs, east-coast Peninsular Malaysia	Mixed HCWs	4 241	BMI ≥ 25 kg/m^2^, height / weight measured; BP measured after 10 min rest; ≥140/90 mmHg classified as hypertension	21.1% obese; 35.1% overweight	Nursing occupation carried almost double the odds of obesity vs. doctors (aOR 1.91, 95% CI 1.45–2.53); Any cardiometabolic comorbidity OR 3.43 (2.71–4.35)
Kaur et al., 2021 [12]	Cross sectional	Sabah	Mixed HCWs	387	BMI Asian cut-offs; DASS-21 for depression; depression / anxiety / stress categorised into normal / mild vs. moderate-to-extremely severe	29% obese	≥ 5 years service aOR 2.23 (1.16–4.28); High depression (DASS-21) aOR 2.08 (1.18–3.71)
Oo et al., 2019 [18]	Cross sectional	Post-basic renal-care nursing students, five intakes	Post basic nursing students	142		Combined 52% overweight / obese	Larger waist circumference; higher SBP/ DBP predicted higher BMI (Wald test)
Sharoni et al., 2023 [19]	Cross sectional	Nurses, tertiary referral hospital	Nurses	417	BMI ≥ 25 kg/m^2^, HPLP-II health behaviour scale	55.9% obese	Nurses: Higher ‘health-responsibility’ (HPLP-II subscale) positively but weakly correlated with BMI (*r* = 0.129, *p* = 0.009); Selected HPLP-II subscales showed variable correlations with BMI
Hong et al., 2019 [20]	Cross sectional	Shift-duty nurses, public teaching hospital	Shift duty Nurses	280	BMI ≥ 25 kg/m^2^; eating behaviour scales; restrained eating style measured and analysed	31.4% obese. 37.1% overweight	Age positively correlated with BMI (*r* = 0.156, *p* < 0.01); Restrained eating style associate with higher BMI (F = 6.06, *p* = 0.003)
Coomerasamy et al., 2014 [21]	Cross sectional	Nurses	Nurses	1086	Self reported BMI ≥ 25 kg/m^2^	33.5% obese	Age ≥ 40 y (vs. < 30 y) Married status ≥ 10 years of service asscoiated with obesity

## Discussion

### Prevalence of overweight and obesity in Malaysia

Across the eight empirical papers reviewed, one-half to two-thirds of Malaysia’s HCWs carry excess body weight. Our point estimates are comparable to European HCW cohorts in similar settings and align with Malaysian HCW estimates [22]. The combined overweight and obesity prevalence among HCWs (49.0% to 69.0%) is more than double the national adult obesity rate of 22.2% reported in NHMS 2023. Point estimates cluster between 49.0% and 69.0% for combined overweight and obesity, with obesity alone ranging from 17.0% in a national survey [21] to 59.0% among military hospital staff [23]. Such figures consistently exceed national adult rates reported by NHMS cycles during corresponding years, underscoring that health sector staff are not immune to the obesogenic forces they counsel patients to avoid.

The increasing prevalence of obesity among HCWs poses a serious threat to the medical profession and to the individuals themselves [24]. The evidence collated in this scoping review shows that work contexts exert a powerful influence on body mass trajectories in Malaysia’s health sector. This study affirms that obesity remains a significant risk even among those working within healthcare settings which may contribute to early morbidity and premature retirement [25]. This review also highlights several variables that are strongly associated with obesity that may generate a greater momentum to encourage HCWs and employer to combat obesity thoroughly. Nursing and other round-the-clock roles repeatedly emerge as high-risk groups, underscoring the metabolic toll of shift work, long spells of standing punctuated by sedentary charting, and scarce access to healthy food during night shifts. Ageing within the profession further compounds risk; staff with longer tenure consistently exhibit greater adiposity, a pattern that mirrors cohort data from other countries and argues for preventive measures early in a HCW’s career. Lifestyle and psychosocial drivers appear to intertwine; low physical activity levels and suboptimal dietary routine coexist with restrained eating tendencies and body image concerns, suggesting that information campaigns alone are unlikely to succeed without supportive environments and stress management opportunities.

### Domain-specific findings

A domain-centred lens helps translate the review findings into a coherent strategic agenda. Accordingly, we categorized them into sociodemographic and occupational influences, behavioural and lifestyle factors, psychosocial and mental health, and anthropometric and biomedical markers. Domains and patterns of risk factors associated with overweight and obesity among HCWs in Malaysia are presented in Table 3.

**Table 3:** Domains and patterns in associated factors associated with obesity

Domain	Risk factor signals	Seen in *n*/8 studies	Findings
Sociodemo-graphic and occupational	Age ≥ 40 years; length of service ≥ 5–10 years; nursing/ support allied health; lower education	7/8	Nurses had almost double the odds of obesity vs. doctors (aOR 1.91, 95% CI 1.45–2.53) (Kaur 2021); Older age and longer tenure associated with higher obesity indices. (Kit 2020).
Behavioural and Lifestyle	Physical inactivity; breakfast skipping and sugary drinks; restrained eating style	2/8	Restrained eating style associated with higher BMI (F = 6.06, *p* = 0.003) (Hong 2019); Higher health responsibility scores showed weak but positive correlations with BMI (*r* = 0.129, *p* = 0.009) (Sharoni 2023).
Psychosical and mental health	Depressive symptoms Sleep <6 h; knowedge-behaviour gap	3/8	High depression score doubled obesity risk (aOR 2.08, 1.18–3.71) (Kaur 2021); Poorer self-rated health predicted higher obesity indices (Kit 2020).
Biomedical / Anthro-pometric	Large waist circumference; elevated SBP/ DBP; cardiometabolic comorbidities	2/8	Every 1 cm rise in waist circumference increased odds of obesity by ~ 7% (Oo 2019) Higer SBP/DBP predicted higher BMI (Oo 2019; Kunyahamu)

*SBP* systolic blood pressure, *DBP* diastolic blood pressure, *BMI* body mass index, *aOR* adjusted odds ratio, *CI* confidence interval

While the reviewed studies collectively highlight the multifactorial nature of obesity among HCWs, critical appraisal of the evidence reveals uneven strength across domains. Most studies adopted cross-sectional designs with small samples and limited adjustment for confounders, restricting causal inference. The sociodemographic and occupational domain was the most frequently investigated (7 of 8 studies), whereas behavioural and lifestyle factors and anthropometric and biomedical markers appeared in only two studies each, and psychosocial and mental health influences in three. This imbalance indicates that several risk domains remain underexplored in the Malaysian HCW context, limiting confidence in some conclusions and the proposed interventions.

The evidence base thus supports a hierarchy of certainty: findings related to age, tenure, and occupation are relatively robust, whereas those concerning behaviour, stress, or mental health are more tentative. Consequently, recommendations should reflect this gradient—interventions targeting lifestyle and workplace structure can be advanced with moderate confidence, whereas those addressing psychosocial risk or emotional regulation should be regarded as exploratory priorities requiring future validation. Acknowledging these evidence gaps provides a more transparent synthesis and strengthens the rationale for future longitudinal and intervention-based research.

#### Sociodemographic and occupational influences point to the importance of work context

Excess weight clusters among mid-career staff particularly nurses and other round-the-clock staffs with risk rising steadily after five to ten years of service [11, 17]. These observations support early career preventive strategies. Orientation programmes for new nurses could include a ‘healthy shift toolkit’ that covers circadian-friendly meal timing, brief resistance-exercise routines and sleep-hygiene tips before maladaptive habits take hold. Protecting a 20-minute meal window within every eight-hour shift and ensuring after-hours access to fresh food would address one of the most frequently cited barriers [26]. To embed accountability, hospital managers could be evaluated on a ward-level wellness index that tracks participation in on-site physical-activity initiatives and adherence to scheduled meal breaks. Given that seven of eight studies reported occupational correlates, these findings carry moderate empirical confidence and justify early implementation of structural workplace interventions.

#### Behavioural and lifestyle factors form the second pillar of intervention

The consistently low physical-activity scores and calorie-dense snacking patterns reported in several studies [18–20] suggest that built-environment nudges [27] represent feasible intervention entry points. Stairwell art, directional lighting and departmental step count competitions can normalise incidental activity, while modest price subsidies in high-fibre night-shift meals, funded by a small ‘sugar surcharge’ on canned drinks can realign café choices with dietary guidance. Being obese greatly increases the risk of developing type 2 diabetes [28], which in turn predisposes individuals to diabetic vascular complications [29]. Behavioural activation tactics at the micro level [30] such as five-minutes stretch-band sessions during shifts handovers have shown promise in corporate settings and could be adapted to clinical wards with minimal disruption. However, because only two studies directly assessed behavioural/lifestyle factors, the strength of evidence for behavioural/lifestyle-based interventions remains limited, and these approaches should therefore be implemented as pilot programmes with embedded evaluation components.

#### Psychosocial and mental health drivers amplify behavioural risk

Only three of the eight studies examined psychosocial or mental health factors, underscoring a major evidence gap. One reviewed study found that depressive symptoms doubled the odds of obesity [12], while others linked chronic sleep restriction (< 6 h) and poor self-rated health to higher BMI [11, 17]. These findings suggest a possible bidirectional pathway between psychological distress and metabolic dysfunction. Recent studies reveal multiple biological mechanisms linking obesity to poor mental health. Obesity-induced cellular senescence disrupts neurogenesis and promotes anxiety and depressive symptoms [31], while hyperactivation of the hypothalamic–pituitary–adrenal axis under metabolic stress heightens stress sensitivity and hormonal imbalance [32]. Yet, because of the limited number and cross-sectional nature of these studies, causality cannot be inferred. Proposed interventions—such as integrated wellness clinics combining weight management and depression/sleep-disorder screening [33] could be considered. A practical model is the integrated wellness clinic that offers a combined BMI check, brief depression screen and immediate referral to a dietitian. In parallel, secure peer-support channel moderated by occupational psychologists can deliver short cognitive behavioural lessons on fatigue management and stress eating [34]. Given limited studies in Malaysia in this domain, strengthening this area through well-designed mixed-methods research will be critical to integrating psychosocial care into workplace obesity-prevention strategies.

#### Anthropometric and biomedical markers signal that cardiometabolic damage is already occurring

Younger cohorts of nurses showed raised blood pressure and central adiposity, foreshadowing future non-communicable disease burden [11, 18]. This pattern can be explained by established pathophysiology that obesity-related hypertension is driven by complex interactions among kidney, metabolic, and neuroendocrine pathways. These include overactivation of the sympathetic nervous system (SNS), stimulation of the renin-angiotensin-aldosterone system (RAAS), changes in adipose-derived cytokines such as leptin, insulin resistance, and both structural and functional alterations in the kidneys [35]. It is estimated that nearly 60.0% to 70.0% of hypertension in adults can be directly attributed to obesity [36]. Installing point-of-care kiosks that provide automatic waist circumference and blood pressure measurements accompanied by personalised QR-linked micro goals would make risk visible and prompt self-regulation. Community pharmacist, who have expressed willingness to extend weight-management services, could conduct ‘metabolic tune-up’ drives, fast-tracking high-risk staff to specialist care [37].

Each intervention should incorporate a pragmatic evaluation arm such as stepped-wedge rollout to address the glaring absence of longitudinal Malaysian data on HCWs’ weight trajectories. Taken together, these measures would transform healthcare facilities from obesogenic microenvironments into exemplars of healthy workplace design, protecting staff well-being and preserving the credibility of health-promotion messages delivered to the wider public. Because anthropometric and biomedical parameters were reported in only two studies, these observations are suggestive but not conclusive, underscoring the need for future research incorporating repeated physiological assessments.

In summary, this domain-based synthesis demonstrates that sociodemographic and occupational factors are well supported, whereas behavioural/lifestyle, psychosocial/mental health, and anthropometric/biomedical domains remain less established. Explicitly linking these evidence disparities to the confidence level of each recommendation enables a more critical and transparent synthesis—ensuring that practice guidance remains proportionate to empirical strength while outlining clear directions for future research.

### Strengths

This review synthesised evidence from eight Malaysian studies on HCWs using a transparent PRISMA workflow and organised results within a pre-specified, practice-facing framework. We prioritised studies with measured anthropometry where available and resolved potential dataset overlap before synthesis. However, interpretation is limited by the predominance of cross-sectional designs, heterogeneity in BMI cut-offs and measurement methods, variability in study populations and settings, inconsistent adjustment for confounding, and incomplete reporting. These differences precluded meta-analysis, may increase the risk of bias, and limit generalisability beyond urban public-sector facilities; publication and language biases are also possible. Future work should include longitudinal or interventional designs, standardised definitions and measurement approaches, and richer anthropometric phenotyping to clarify causal pathways and inform targeted workplace interventions.

### Limitations

The evidence synthesised in this review is subject to several limitations. Most included studies employed cross-sectional designs, which limit the ability to infer causality between risk factors and obesity. Furthermore, many studies relied solely on BMI as a proxy for adiposity, which does not capture differences in muscle mass, fat distribution, or bone structure [38]. Such reliance may lead to misclassification and underestimate the risk of central obesity. Additionally, metabolic-risk assessments, such as fasting blood glucose, were often based on a single measurement, potentially limiting the accuracy of metabolic risk estimation [39]. These methodological constraints should be considered when interpreting the findings, and future research would benefit from longitudinal designs, more comprehensive adiposity measures, and repeated anthropometric assessments.

### Implications for practice and policy

Despite its limitations, the synthesis provides actionable insights for intervention planning within Malaysia’s healthcare system. Evidence most consistently supports sociodemographic and occupational interventions—for example, embedding structured meal and rest breaks, protecting early-career staff from shift overload, and incorporating wellness metrics into departmental performance indicators. These areas carry the strongest empirical backing and can be pursued with moderate confidence. Interventions targeting behavioural and psychosocial determinants, although promising, should be introduced as pilot or phased programmes with integrated evaluation components. This may include workplace campaigns promoting incidental activity, healthier cafeteria options, brief mental-health awareness training, and confidential mental-health screening linked to dietetic counselling. Because only a minority of studies examined mental-health correlates, such initiatives should initially emphasise feasibility and acceptability rather than presumed effectiveness. From a policy perspective, the findings highlight the need for institutional alignment between occupational health, hospital management, and human-resource divisions to address obesogenic environments systematically. Regular surveillance of HCW weight status, job strain, and health-behaviour indicators should be institutionalised through annual health-risk assessments. Embedding these components within national occupational-health key performance indicators would strengthen accountability and ensure sustainability.

Collectively, the strengths and limitations of current evidence indicate a mature but still fragmented research field. Establishing longitudinal, multi-centre datasets and integrating psychosocial and organisational metrics will be crucial to move from correlation or association toward causation, and from descriptive mapping to tested intervention efficacy.

### Future research direction

Further longitudinal research is needed to examine causal pathways and the effectiveness of workplace-based interventions. Future studies should incorporate more accurate adiposity measures (e.g., body fat percentage, waist-to-hip ratio) and consider psychosocial and organisational determinants in greater depth.

## Conclusion

This scoping review synthesised evidence on the prevalence and associated factors of overweight and obesity among HCWs in Malaysia. The findings indicate that overweight and obesity risk is shaped by a multifaceted interaction of sociodemographic, behavioural, psychosocial, and anthropometric factors, with sociodemographic and occupational influences being the most frequently reported. Future research should adopt longitudinal designs, incorporate more accurate adiposity a metabolic measure and evaluate the effectiveness of targeted workplace health interventions. Collaborative action from healthcare institutions, policymakers, and occupational health teams will be vital in tackling overweight and obesity among HCWs and fostering a healthier, more resilient workforce in Malaysia.

## Data Availability

All data underlying this article are available in the published studies included in the review and in the supplementary materials.

## Abbreviations

Ov: Overweight
Ob: Obesity
BMI: Body mass index
HCWs: Healthcare workers
